# Evolutionary history of mammalian sucking lice (Phthiraptera: Anoplura)

**DOI:** 10.1186/1471-2148-10-292

**Published:** 2010-09-22

**Authors:** Jessica E Light, Vincent S Smith, Julie M Allen, Lance A Durden, David L Reed

**Affiliations:** 1 Department of Wildlife and Fisheries Sciences, Texas A&M University, College Station, Texas 77843, USA; 2 Florida Museum of Natural History, University of Florida, Gainesville, Florida 32611, USA; 3 Entomology Department, Natural History Museum, London SW7 5BD, UK; 4 Department of Biology, University of Florida, Gainesville, Florida 32611, USA; 5 Department of Biology, Georgia Southern University, Statesboro, Georgia 30460, USA

## Abstract

**Background:**

Sucking lice (Phthiraptera: Anoplura) are obligate, permanent ectoparasites of eutherian mammals, parasitizing members of 12 of the 29 recognized mammalian orders and approximately 20% of all mammalian species. These host specific, blood-sucking insects are morphologically adapted for life on mammals: they are wingless, dorso-ventrally flattened, possess tibio-tarsal claws for clinging to host hair, and have piercing mouthparts for feeding. Although there are more than 540 described species of Anoplura and despite the potential economical and medical implications of sucking louse infestations, this study represents the first attempt to examine higher-level anopluran relationships using molecular data. In this study, we use molecular data to reconstruct the evolutionary history of 65 sucking louse taxa with phylogenetic analyses and compare the results to findings based on morphological data. We also estimate divergence times among anopluran taxa and compare our results to host (mammal) relationships.

**Results:**

This study represents the first phylogenetic hypothesis of sucking louse relationships using molecular data and we find significant conflict between phylogenies constructed using molecular and morphological data. We also find that multiple families and genera of sucking lice are not monophyletic and that extensive taxonomic revision will be necessary for this group. Based on our divergence dating analyses, sucking lice diversified in the late Cretaceous, approximately 77 Ma, and soon after the Cretaceous-Paleogene boundary (ca. 65 Ma) these lice proliferated rapidly to parasitize multiple mammalian orders and families.

**Conclusions:**

The diversification time of sucking lice approximately 77 Ma is in agreement with mammalian evolutionary history: all modern mammal orders are hypothesized to have diverged by 75 Ma thus providing suitable habitat for the colonization and radiation of sucking lice. Despite the concordant timing of diversification events early in the association between anoplurans and mammals, there is substantial conflict between the host and parasite phylogenies. This conflict is likely the result of a complex history of host switching and extinction events that occurred throughout the evolutionary association between sucking lice and their mammalian hosts. It is unlikely that there are any ectoparasite groups (including lice) that tracked the early and rapid radiation of eutherian mammals.

## Background

Lice (Insecta: Phthiraptera) are obligate, permanent ectoparasites of birds and mammals, entirely dependent upon their vertebrate hosts for survival. Four phthirapteran suborders are recognized: the chewing louse suborders Amblycera, Ischnocera, and Rhynchophthirina, and the sucking louse suborder Anoplura [1]. As a group, chewing lice parasitize birds and mammals, and all have chewing mouthparts that they use to feed upon the skin (feathers, fur, and dander) and sometimes the blood of their hosts [2]. Sucking lice, in contrast, parasitize only eutherian mammals and they are morphologically adapted for life on their mammal hosts: they are wingless, dorso-ventrally flattened, possess adaptive tibio-tarsal claws that are used to cling to host hair, and have modified piercing mouthparts for feeding. These ectoparasitic insects are one of only a handful of haematophagous arthropod groups that use their highly derived mouthparts to feed directly from host blood vessels [3].

The blood-feeding habits of sucking lice and the close association they have with their mammalian hosts are hypothesized to have evolved via a particular sequence of events [4-6]. Early in their evolutionary history, sucking louse ancestors had simple chewing mouthparts and were free-living associates of the nests and burrows of vertebrates. These nests and burrows served as protective habitats as well as a source of unlimited food supplies such as fungi, dung, and organic debris, specifically sloughed skin, fur, and feathers [5-7]. Over time, some of these nest associates became more directly dependent on their hosts and transitioned from opportunistic associates to obligate parasites. These parasitic species fed directly from their hosts, ingesting more nutritious and easier to digest blood (compared to organic debris such as sloughed skin and feathers) and subsequently developed specialized mouthparts modified to obtain blood meals [2,7]. This succession of events from free-living nest associates to obligate parasites can be seen by examining the closest living relatives of the Anoplura, members of other phthirapteran suborders, and the bark and book lice (order Psocoptera). The Psocoptera are closely related to the Phthiraptera (together they form the superorder Psocodea), and these non-parasitic insects often interact with vertebrate taxa, living in the nests, burrows, or among the fur and feathers of mammals and birds and use their chewing mouthparts to feed on fungi or organic matter [7-10]. Within the Phthiraptera, phylogenetic studies have shown sucking lice to be monophyletic and derived, nested within the chewing lice and sister to the Rhynchophthirina, a small suborder of chewing lice (3 known species) parasitic on warthogs, bush pigs, and elephants [1,11-13]. Rhynchophthirina species have modified chewing mouthparts attached to the end of a long proboscis that are used to break through the skin of their hosts allowing pools of blood to form. These chewing lice then use their mouthparts to feed on the blood collected in these pools. Thus, it is likely that sucking lice evolved from a blood-feeding Rhynchophthirina-like ancestor with the highly modified anopluran mouthparts derived from the ancestral chewing mouthparts found in all other lice [14-16].

**Figure 1:**
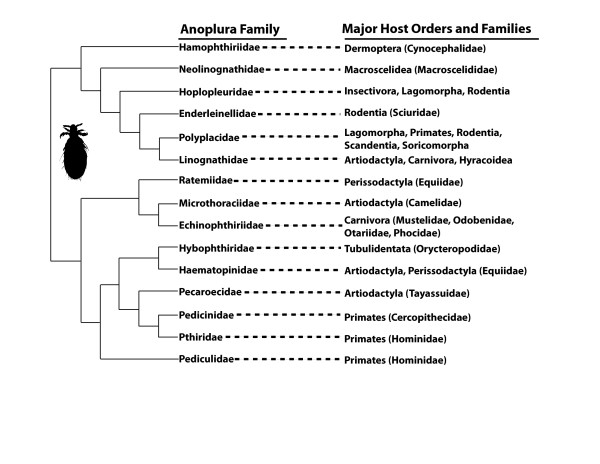
**Hypothesized relationships among Anoplura families (and their host associations) redrawn from Kim **[22].

When sucking lice began their associations with mammals is uncertain because fossil evidence within the Phthiraptera is generally lacking [10,17,18]. Psocopteran groups are hypothesized to have originated in the Mesozoic Era [5,10,18,19], with dates ranging from the Cretaceous (65 Million Years Ago; Ma) as far back to the Permian (260 Ma) for the origin of the Phthiraptera [2,4,5,10,18,19]. However, the recent discovery of two important fossils has shed light on the age of lice. The first is an exceptionally preserved 44 Ma bird louse fossil [17], and the second is a 100 Ma fossil of the book-louse family Liposcelididae [18], which is the closest free-living relative of parasitic lice [13]. These two fossils imply a rather ancient origin of lice and therefore it is reasonable to assume that given their restricted host associations, parasitic lice originated on their vertebrate hosts [5]. Most placental mammalian orders had originated by the end of the Cretaceous, 85-100 Ma [20], thus providing suitable habitats for sucking lice to colonize. Since their origination, sucking lice have successfully diversified and there now are more than 540 described species of Anoplura worldwide that can be assigned to 50 genera in 15 families [21-24]. Sucking lice parasitize members of 12 of the 29 recognized mammalian orders, and are generally host-specific with families, genera, and species of lice parasitizing closely related hosts (Figure 1 and Table 1). Of the non-parasitized potential host taxa, 11 mammalian orders are not known to be parasitized by any louse species, whereas representatives of the remaining six orders (Dasyuromorphia, Didelphimorphia, Diprotodontia, Paucituberculata, Proboscidea, and Pilosa) are parasitized only by chewing lice. Although cospeciation is perceived to be common between parasitic organisms and their hosts, given the current host associations of sucking lice (Figure 1 and Table 1) it is unlikely that parallel evolution has been the dominant process shaping the radiation of this assemblage. Rather, it is probable that the associations between sucking lice and their eutherian hosts involves a complex history of multiple colonization events and small bouts of cospeciation, colonization failures, extinction events, and host switches across eutherian lineages [6,22,25].

**Table 1:** Generalized Anoplura host associations*

Louse Family	Louse Genus (Number of Species)	Common Names of Major Host Groups (Host Orders)
Echinophthiriidae	Antarctophthirus (6)	Seals, Walrus (Carnivora)
	Echinophthirius (1)	Earless Seals (Carnivora)
	Latagophthirus (1)	Otters (Carnivora)
	Lepidophthirus (2)	Earless Seals (Carnivora)
	Proechinophthirus (2)	Sea Lions (Carnivora)

Enderleinellidae	Atopophthirus (2)	Giant Flying Squirrels (Rodentia)
	Enderleinellus (45)	Squirrels (Rodentia)
	Microphthirus (1)	Flying Squirrels (Rodentia)
	Phthirunculus (1)	Giant Flying Squirrels (Rodentia)
	Werneckia (5)	Squirrels (Rodentia)

Haematopinidae	Haematopinus (21)	Artiodactyls, Equids (Artiodactyla, Perissodactyla)

Hamophthiriidae	Hamophthirius (1)	Colugos (Dermoptera)

Hoplopleuridae	Ancistroplax (5)	Shrews (Soricomorpha)
	Haematopinoides (1)	Moles (Soricomorpha)
	Hoplopleura (141)	Rodents, Pikas (Rodentia, Lagomorpha)
	Paradoxophthirus (1)	Asian Rock Squirrel (Rodentia)
	Pterophthirus (5)	Spiny Rats (Rodentia)
	Schizophthirus (9)	Dormice (Rodentia)

Hybophthiridae	Hybophthirus (1)	Aardvarks (Tubulidentata)

Linognathidae	Linognathus (52)	Artiodactyls (Artiodactyla), Canids (Carnivora)
	Prolinognathus (8)	Hyraxes (Hyracoidea)
	Solenopotes (9)	Bovids, Cervids (Artiodactyla)

Microthoraciidae	Microthoracius (4)	Camels, Lamas (Artiodactyla)

Neolinognathidae	Neolinognathus (2)	Elephant Shrews (Macroscelidea)

Pecaroecidae	Pecaroecus (1)	Peccaries (Artiodactyla)

Pedicinidae	Pedicinus (14)	New World Primates (Primates)

Pediculidae	Pediculus (3)	Old World Primates (Primates)

Polyplacidae	Abrocomaphthirus (2)	Chinchilla Rats (Rodentia)
	Ctenophthirus (1)	Spiny Rats (Rodentia)
	Cuyana (1)	Chinchillas (Rodentia)
	Docophthirus (1)	Tree Shrews (Scandentia)
	Eulinognathus (27)	Rodents (Rodentia)
	Fahrenholzia (12)	Heteromyid Rodents (Rodentia)
	Galeophthirus (1)	Cavies (Rodentia)
	Haemodipsus (7)	Rabbits and Hares (Lagomorpha)
	Johnsonpthirus (5)	Squirrels (Rodentia)
	Lagidophthirus (1)	Chinchillas (Rodentia)
	Lemurpediculus (2)	Dwarf Lemurs (Primates)
	Lemurphthirus (3)	Bush Babies (Primates)
	Linognathoides (11)	Squirrels (Rodentia)
	Mirophthirus (1)	Pygmy Dormice (Rodentia)
	Neohaematopinus (31)	Squirrels, Murids (Rodentia)
	Phthirpediculus (3)	Lemurs (Primates)
	Polyplax (78)	Rodents, Shrews (Rodentia, Soricomorpha)
	Proenderleinellus (1)	Pouched Rats (Rodentia)
	Sathrax (1)	Tree Shrews (Scandentia)
	Scipio (3)	Cane, Dassie Rats (Rodentia)
	Typhlomyophthirus (1)	Pygmy Dormice (Rodentia)

Pthiridae	Pthirus (2)	Old World Primates (Primates)

Ratemiidae	Ratemia (3)	Equids (Perissodactyla)

*Host associations and number of louse species are based on Durden and Musser [23,24] and recent publications.

To date, there have been only two studies that have attempted to reconstruct phylogenetic relationships among anopluran families [21,22]. Kim and Ludwig [21] studied 15 taxa based on 22 morphological characters whereas Kim [22] examined 47 taxa with 39 morphological characters. Both studies were based on small morphological datasets with few characters, the phylogenetic utility of which has been questioned [10]. While there have been multiple studies examining anopluran relationships within genera and among apparently closely related genera and families [26-29], a higher-level phylogeny of sucking lice is lacking. This study is the first to use molecular data and estimates of divergence times to elucidate the evolutionary history of this unique haematophagous group in relation to their mammalian hosts.

## Results

### Taxon Sampling, Data Collection, and Phylogenetic Analyses

Lice were obtained from 8 of the 15 sucking louse families (Additional File 1). Unfortunately, louse data from the remaining 7 families could not be obtained due to specimen rarity (some anopluran families have extremely narrow host ranges and some are monotypic, making collection almost impossible) or failure to amplify specimens in the laboratory. Because of PCR failure as well as availability of data from GenBank, some molecular data could not be collected. The genes 18S, EF-1α, and COI were not collected from 11, 7, and 2 specimens, respectively, and, except for one sample of *Pedicinus pictus *(*Pedicinus pictus *2), none of the specimens analyzed were missing data from more than one molecular marker (Additional File 1).

For each gene examined, phylogenetic analyses (MP, ML, and Bayesian) yielded similar topologies, although nodal support and placement of outgroup taxa varied depending on the gene (See Additional Files 2, 3, and 4). Previous studies have noted that the third codon positions of the mitochondrial COI gene tend to be saturated and homoplasious [13]. Saturation plots supported slight saturation of third positions in the COI data analyzed herein; however, phylogenetic analyses including and excluding third codon positions did not result in significant differences to tree topologies or branch lengths with the exception of placement of some of the outgroup taxa (data available upon request). The three genes used in this study were phylogenetically informative at different areas of the phylogeny, similar to previous findings [28]. With 58, 126, and 220 parsimony informative sites for 18S, EF-1α, and COI, respectively, the nuclear genes (18S and EF-1α) provided slightly more resolution basally whereas the mitochondrial marker, COI, provided more resolution at terminal nodes (See Additional Files 2, 3, and 4). Although basal resolution was generally lacking and there were overall differences in resolution for each gene, topologies resulting from phylogenetic analyses of individual genes were not in strong conflict.

**Figure 2:**
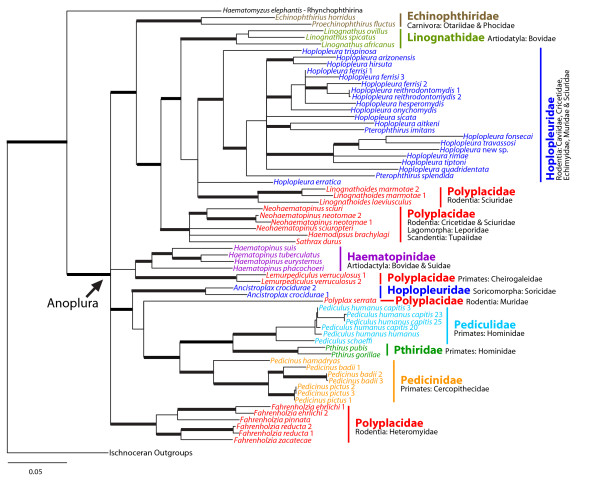
**Bayesian phylogram of the Anoplura based on molecular data**. This Bayesian phylogram is the result from the analysis of the combined 3-gene data set partitioning the data by gene and by codon for the protein coding genes COI and EF-1α. Bayesian posterior probability greater than 0.95 and likelihood support values greater than 75 are indicated by the heavy branches. Taxon names correspond to Additional File 1 and taxon colors correspond to louse family. Louse family and host associations are indicated to the right of each clade. A monophyletic Anoplura is indicated by the arrow.

Analysis of the 3-gene data set using MP, ML, or Bayesian approaches (including BEST and BEAST) resulted in similar topologies, nodal support, and branch lengths (Bayesian phylogram shown in Figure 2). Analyses of the 3-gene data sets seemed to merge different levels of phylogenetic information from each gene resulting in a more resolved phylogeny overall. Bayes factors indicated that partitioning by gene and by codon within each protein coding gene was preferred over a more simple or non-partitioned scheme. Although highly partitioned data sets tend to be preferred according to Bayes factors [30], partitioned and non-partitioned analyses yielded similar topologies and support values. The species tree constructed with BEST lacked resolution but did not conflict with individual gene trees or trees resulting from concatenated and partitioned phylogenetic analyses. Although there were some topological differences depending on the analysis and partitioning scheme, these differences always involved clades that were not strongly supported. One topological difference that appeared in some phylogenetic analyses was the placement of the mouse louse *Polyplax serrata*. Depending on the data set examined (individual genes, 3-gene data set, etc.) and phylogenetic method, topological placement of *P. serrata *varied from being closely related to primate lice (Figure 2) to located near the base of the tree (See Additional Files 3 and 4). However, the phylogenetic position of *P. serrata *never received substantial support, and exclusion of this taxon from phylogenetic analyses resulted in topologies that were not in conflict with the phylogeny shown in Figure 2.

All phylogenetic trees reconstructed in our analyses support a monophyletic Anoplura, sister to the chewing louse suborder Rhychophthirina (Figure 2). Several anopluran families (Hoplopleuridae and Polyplacidae), genera (*Hoplopleura *and *Pterophthirus*), and species (*H. ferrisi*) were not monophyletic. Phylogenetic constraints forcing the families and genera to be monophyletic were significantly worse than the best tree (ML Shimodaira-Hasegawa tests and examination of Bayesian suboptimal trees; *P *< 0.05). The remaining louse families were all monophyletic, although it is important to note that taxon sampling for many of these groups was low (Figure 2 and Additional Files 2, 3, and 4). Notably, primate lice (excluding the polyplacid louse *Lemurpediculus verruculosus*) belonging to the anopluran families Pedicinidae, Pediculidae, and Pthiridae formed a highly supported monophyletic group (Figure 2).

For the most part, lice did not form monophyletic groups according to host associations. There are two clades of lice parasitizing artiodactyl mammals (*Linognathus *and *Haematopinus*, belonging to the families Linognathidae and Haematopinidae, respectively), probably resulting from two independent colonization events of the hosts (Figure 2). Lice parasitizing rodents were far from monophyletic, and instead were scattered throughout the phylogeny in five distinct clades (Figure 2). One rodent-louse clade containing the polyplacid genus *Neohaematopinus *also included the lice *Haemodipsus *and *Sathrax*. These two louse genera do not parasitize rodents and instead are associated with rabbits and hares, and tree shrews, respectively. Within Rodentia, lice parasitizing the families Cricetidae, Muridae, and Sciuridae also were not monophyletic (Figure 2). Sciurid lice (*Hoplopleura*, *Linognathoides*, and *Neohaematopinus*) are distributed among three clades and cricetid (*Hoplopleura *and *Neohaematopinus*) and murid (*Polyplax *and *Hoplopleura*) lice are each distributed across two clades. Some host lineages, however, were parasitized by monophyletic lineages of lice. These host groups include heteromyid rodents (parasitized by the louse genus *Fahrenholzia*), carnivores (although the sample size of lice parasitizing carnivores was extremely small), and primates (except Cheirogaleidae; Figure 2).

### Estimates of Divergence Times

**Figure 3:**
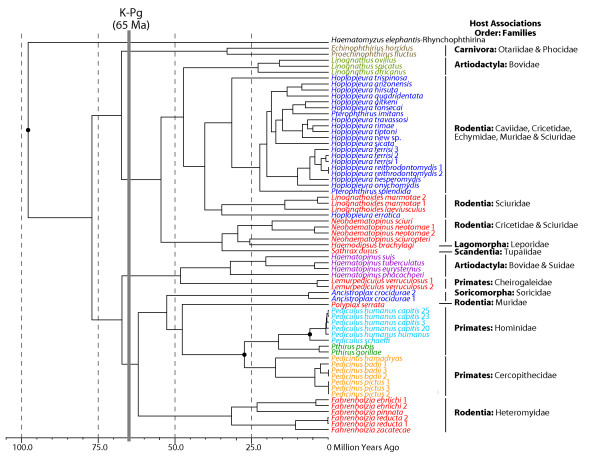
**Chronogram for the Anoplura**. Shown is the Bayesian topology resulting from analysis of the 3-gene data set (partitioning the data by gene and by codon for the protein coding genes COI and EF-1α) in BEAST [36], which differs only slightly from the topology shown in Figure 2 (there is weak support for these differences; see text). Divergence times were estimated using three calibrations (94-101 Ma for the spilt between Rhynchophthirina and Anoplura, 20-25 Ma for the split between Old World Monkey lice and hominoid lice, and 5-7 Ma for the split between human and chimpanzee-associated *Pediculus *lice), indicated by filled circles at nodes. Taxon colors correspond to louse family indicated in Figure 2 and host associations are indicated to the right of each clade. The Cretaceous-Paleogene (K-Pg) boundary is indicated at 65 Ma by the dark gray vertical bar. Upper and lower bounds of the 95% highest posterior density interval (95% HPD) for each node are available in Additional File 5.

The molecular clock was rejected in the combined 3-gene data set; thus, the most appropriate divergence dating techniques are those that relax a molecular clock [31]. Similar to phylogenetic analyses, Bayes factors indicated that partitioned data sets are the preferred partitioning scheme, although analyses of partitioned and non-partitioned data sets produced similar results. Partitioned analyses (with model parameters unlinked across partitions) using all three calibrations resulted in a late Cretaceous origin of the Anoplura, and a time of basal diversification approximately 77 Ma (95% HPD 58-96 Ma; Figure 3 and Additional File 5). Upon initially parasitizing their eutherian hosts, the Anoplura segregated into two clades and then diversified rapidly soon after the Cretaceous-Paleogene (K-Pg) boundary approximately 65 Ma (Figure 3). In one clade (the top clade in Figure 2), sucking lice radiated to parasitize carnivores, artiodactyls, rodents, rabbits, and tree shrews, and in the other clade (the bottom clade in Figure 2), anoplurans diverged to colonize artiodactyls, shrews, rodents, and primates. There appears to be no evidence for parallel cladogenesis between sucking lice and their hosts early in their evolutionary history. Rather, it seems that these parasitic insects independently colonized diverse mammal groups possibly as these host lineages were radiating.

## Discussion

### Anoplura Phylogeny

This study represents the first phylogenetic hypothesis of sucking louse relationships using molecular data. The genes selected for use in this study (18S, EF-1α, and COI) have proven phylogenetically informative for lice in other studies [1,11-13,27,28,32]. However, even when used in combination, these genes fail to completely resolve relationships at a higher level across Anoplura, which is not unexpected given the relatively small number of species sampled in this study. There is good support for many of the louse lineages, but it is unclear how these major lineages are related because some branches are short and lack support (Figure 2). Future studies will need to increase taxon sampling as well as include additional molecular markers to better resolve phylogenetic relationships among sucking louse species.

The mitochondrial gene COI has been a staple in louse phylogenetic work (see references above); however, rapid rates of evolution and data saturation in this marker are a concern especially for higher-level studies [13,26]. Sequence divergence within a louse morpho-species can be rather high, upwards to 15% uncorrected p-distances [27,32,33], and we found similar results in this study. For example, the two *Ancistroplax crocidurae *specimens were 13.5% divergent, the *Lemurpediculus verruculosus *specimens were 14.7% divergent, and the *Linognathoides marmotae *specimens were 12.5% divergent (all uncorrected p-distances). While these divergences may be indicative of cryptic species, it is more likely that these high numbers for the COI gene are typical for louse lineages because sequences for the nuclear markers were identical. While COI saturation was not an obvious problem in the current study, the phylogenetic placement of *Polyplax serrata *appears to be the result of elevated rates of evolution in this mitochondrial marker (See Figure 2 and Additional Files 3 and 4). Future studies may encounter similar problems; therefore, inclusion of other data would be helpful when attempting to resolve higher-level relationships among lice. These additional data could be molecular or morphological (see below); however, finding appropriate molecular markers for use within Phthiraptera has often been difficult [28] and it is likely that novel approaches will be necessary to resolve phylogenetic relationships in this insect group.

Unfortunately, not all anopluran families could be included in this study, resulting in an incomplete picture of phylogenetic relationships in this louse lineage. To better elucidate the evolutionary history of sucking lice, it is imperative that future studies increase the diversity of taxon sampling (in addition to utilizing additional data; see above). Even with incomplete taxonomic sampling, a few aspects of anopluran systematics are apparent, specifically the lack of monophyly of the families Hoplopleuridae and Polyplacidae (Figure 2). This lack of monophyly is not surprising; Hoplopleuridae and Polyplacidae are the two largest sucking louse families, with 162 and 193 described species, respectively (Table 1). Representing such a large number of species, it is likely that these two louse families are taxonomic hodgepodges in need of additional examination and substantial revision. Revision of these two families (as well as genera within both families) will not be possible without more complete taxon sampling. At a lower taxonomic level, the results presented in the current study are in agreement with previous research that focused on smaller anopluran groups [27-29,34].

Currently, the morphology-based classification of Anoplura detailed by Kim and Ludwig [21] and with modifications by Durden and Musser [23] is followed by most researchers studying sucking lice. The molecular phylogeny reported here (Figure 2) agrees with aspects of this morphology-based classification such as the distinct familial lineages of Anoplura associated with pinnipeds (anopluran family Echinophthiriidae), bovids (Linognathidae), bovids and suids (Haematopinidae), hominids (Pediculidae and Pthiridae) and cercopithecids (Pedicinidae). The differences between the two phylogenies (compare Figures 1 and 2) are intriguing; however, it is possible that morphological features may support some of the molecular-based relationships proposed here. For example, the molecular phylogeny places the hoplopleurid genus *Pterophthirus *within the genus *Hoplopleura *(Figure 2). Morphologically, the only difference between these two genera is the extension, to varying degrees, of the second pair of paratergal plates on the abdomen. Perhaps the varying extensions of the second pair or paratergal plates evolved more than once within *Hoplopleura *and, as such, it may not warrant the recognition of *Pterophthirus *as a distinct genus [35]. The wide separation between hoplopleurid genera *Hoplopleura *(including *Pterophthirus*) and *Ancistroplax *in the molecular phylogeny (Figure 2) also has morphological ramifications. Kim and Ludwig [21] recognized two subfamilies within the Hoplopleuridae, the only anopluran family for which they recognized subfamilies. Members of the subfamily Hoplopleurinae (genera *Hoplopleura, Pterophthirus*, and *Paradoxophthirus*) have a large continuous sternite on abdomonal segment 2 that physically connects with the corresponding paratergal plates, situated laterally. However, in members of the subfamily Haematopinoidinae (genera *Ancistroplax, Haematopinoides*, and *Schizophthirus*), the abdominal segment two sternite is clearly divided medially resulting in two separate plates. It is feasible that this morphological difference actually defines two distinct families rather than subfamilies as supported by the large separation between the two clades in Figure 2. As such, it would be beneficial for future researchers to include other relevant hoplopleurid genera in their molecular phylogenetic reconstructions of Anoplura evolutionary history to determine if the two Hoplopleura subfamilies remain genetically distinct.

The most obvious differences between the morphological (Figure 1) and molecular (Figure 2) anopluran phylogenies involve the family Polyplacidae, which is monophyletic based on morphological data but paraphyletic based on molecular data. Interestingly, the morphological definition of the Polyplacidae is quite variable. Notwithstanding the features they share with all other anopluran families, the only morphological characters that are common to all members of the Polyplacidae, as currently recognized, are the presence of 5 antennal segments, 6 pairs of spiracles on the abdomen, small forelegs, and the absence of a notal pit on the thorax [21]. However, none of these characters are synapomorphies for Polyplacidae. Statements reflecting the morphological variability of Polyplacidae in current descriptions include: "antennae...usually sexually dimorphic," "thorax with mesothoracic phragma usually present," "abdomen with paratergites usually highly developed...and occasionally represented by small sclerites or completely lacking," "tergal and sternal plates usually highly developed and at times reduced or lacking," "male...with variously shaped basal apodeme, parameres and pseudopenis," and "female with...spermatheca usually indistinct" [21]. This extreme morphological variability within the Polyplacidae may actually encompass more than one family as suggested by the separate polyplacid lineages shown in Figure 2. The molecular data suggest that rigorous taxonomic reassessment of what is currently treated as Polyplacidae is warranted. In fact, all five of the separate polyplacid lineages shown in Figure 2 correspond with distinct morphological characters that could be used to define separate families and other suprageneric taxa if future taxonomic reevaluation supports such action.

Without complete taxonomic sampling at the family level, it is difficult to compare Kim's [22] morphological hypothesis of anopluran relationships (Figure 1) to the molecular phylogeny (Figure 2). A few additional differences, however, are apparent. For one, morphological data support a sister relationships between Pedicinidae and Pthiridae, and these two louse families are closely related to Pecaroecidae, Haematopinidae, and Hybophthiridae, all to the exclusion of Pediculidae [22]. Molecular data, however, support monophyly of all non-polyplacid primate lice (families Pedicinidae, Pediculidae, and Pthiridae) with a relatively distant relationship to the Haematopinidae (Figure 2). Monophyly of these three primate louse families has been found in previous molecular studies [28,29]; however, molecular data from the families Hybophthiridae, and Pecaroecidae will be needed for a more rigorous comparison to the morphological study of Kim [22]. Interestingly, Kim [22] noted a relatively close relationship among the families Hoplopleuridae, Linognathidae, and Polyplacidae (Figure 1). The molecular data presented herein also support a close relationship among these three families (or at least specific clades within Hoplopleuridae and Polyplacidae; Figure 2) and it will be interesting to see if these relationships hold with additional investigations. Although a more comprehensive morphological study is currently underway (Smith and Light, unpubl. data), additional molecular data and better taxon sampling will be necessary to properly compare morphology and molecules and it is likely that these data will both agree that substantial taxonomic revision of Anoplura will be necessary.

### Host Associations and the Origin of Anoplura

Using a Bayesian approach implemented in the program BEAST v1.5.3 [31,36], we estimated a late Cretaceous diversification of sucking lice (approximately 77 Ma; Figure 3 and Additional File 5). In this analysis, we utilized the 3-gene data set and three calibration points, and we allowed substitution and clock models to be unlinked. Divergence time estimates varied, however, if clock models or clock and substitution models were linked across the three data partitions, and when calibration points were not used concurrently or used as hard bounds. When clock models or clock and substitution models were linked across the three data partitions, estimates of divergence times tended to be much more recent, with an Eocene or Paleocene origin of the Anoplura, approximately 55 Ma. However, linking substitution or clock models across partitioned data sets is the equivalent of performing analyses on concatenated data sets (i.e., not applying different models of evolution to each partition). Since Bayes Factors comparisons performed herein support partitioned analyses, we concentrate our discussion below on the estimates obtained with unlinked model parameters.

Of our three calibration points, one was located basally whereas the other two were located more terminally on the louse phylogeny (Figure 3). Previous studies have found that the use of single calibration points, especially when placed either basally or terminally, can result in erroneous estimations of divergence times [28,37-39]. Similarly, our analyses using only the basal calibration resulted in overestimates of ages at terminal nodes and analyses using only the terminal primate-louse calibrations resulted in underestimates of the ages at basal nodes. In fact, these over- and underestimates were extreme, 63 Ma for the split between Old World and hominoid-associated lice, and 33 Ma for the age of Anoplura, respectively. Other studies have found that use of hard bounds is often ill-advised (especially when there is a lack of confidence of the exact ages of the calibration) and that fossils provide poor hard maximum bounds [38,40,41]. Analyses using only upper bounds also resulted in underestimates for the age of Anoplura, thus providing additional support for the simultaneous use of the three calibration points. Further analyses incorporating increased taxon sampling and additional calibrations (if available) will be necessary to test the hypotheses presented here.

Because the fossil record for lice is so poor [10,17,18,42], our calibrations were based on well-documented cospeciation events between these parasites and their mammalian hosts. The primate-louse calibrations have been used in several previous studies [28,29] and have generally proven useful to better understand louse evolutionary history. For a basal calibration representing the split between Rhynchophthirina and Anoplura, we chose the time of basal diversification in placental mammals from Bininda-Emonds et al. [20] because we believed it reasonable that sucking lice could not have diversified until they had appropriate hosts (i.e., placental mammals) to colonize [5,20]. Additionally, the recent discovery of two louse fossils at 44 Ma [17] and 100 Ma [18] adds further weight to support an ancient origin of lice, as does results of a recent study examining diversification times across all suborders of lice utilizing these fossils. This study found that sucking lice diversified approximately 75 Ma [Smith VS, Ford T, Johnson KP, Johnson PCD, Yoshizawa K, Light JE: Multiple lineages of lice pass through the K-Pg boundary, Submitted]. This result is similar to our own findings, further supporting use of a basal calibration point of 94-109 Ma. It is important to note that because some of the outgroup taxa used in this study (e.g., *Bovicola, Felicola*, and *Neotrichodectes*) also parasitize eutherian mammals, the Bininda-Emonds et al. [20] calibration could have been placed at the root of the phylogeny. However, these chewing lice are recently derived lineages within Ischnocera and likely radiated as a result of a recent host switch to eutherian mammals [43].

**Figure 4:**
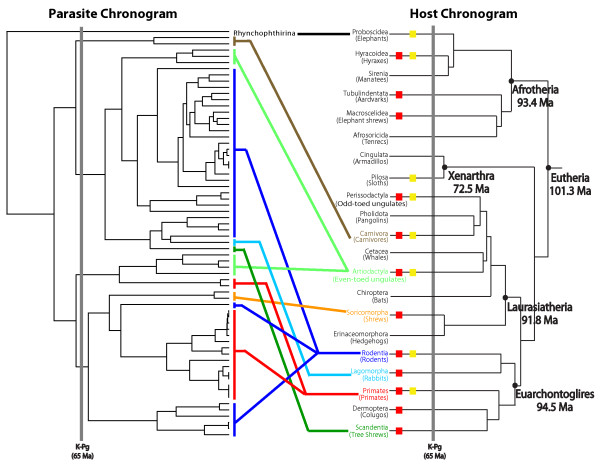
**Comparison of host and parasite chronograms**. The parasite chronogram is redrawn from Figure 3 and the host chronogram (with dates of major mammalian divergences) is redrawn from Bininda-Emonds et al. [20]. Lines drawn between taxa indicate host-parasite associations. On the host chronogram, lineages with red and yellow boxes are parasitized by sucking lice and chewing lice, respectively. Mammalian lineages without shaded boxes are not known to be parasitized by any louse group. The Cretaceous-Paleogene (K-Pg) boundary indicated at 65 Ma by the dark gray vertical bar.

Soon after colonizing their hosts, sucking lice appear to have diversified rapidly, parasitizing multiple mammalian orders and families soon after the Cretaceous-Paleogene (K-Pg) boundary, approximately 65 Ma (Figure 3). Two major louse clades formed relatively early in anopluran evolutionary history, and these two clades parasitize members of a diverse assortment of mammal orders: one louse clade parasitizes Artiodactyla, Carnivora, Lagomorpha, Rodentia, and Scandentia, whereas the other clade parasitizes Artiodactyla, Primates, Rodentia, and Soricomorpha (Figure 3). These host groups are often distantly related, and thus the louse phylogeny bears little similarity to the host tree (Figure 4). Furthermore, several mammal groups, specifically artiodactyls, rodents, and primates, are parasitized by multiple, distantly related louse lineages (Figures 3 and 4). In fact, the majority of Anoplura parasitize these host groups (approximately 90%; Table 1). Thus, it is likely that the associations between sucking lice and eutherian mammals are the result of a complex history of host switching and extinction events both early and late during their evolutionary history. Host switching has been documented in the literature for sucking lice [44] as well as other ectoparasites [45-47]. In this study, clear instances of recent host switching include *Lemurpediculus *parasitizing mouse lemurs and *Ancistroplax *parasitizing shrews. These two louse genera originated 5-10 Ma and their hosts, in contrast, diverged 35 Ma (Cheirogaleidae) and 50 Ma [20], respectively. Cospeciation, however, also has played a significant role in shaping associations between sucking lice and their mammalian hosts, especially at lower taxonomic levels. Examples include squirrels and their lice [22,25], primates and lice belonging to the families Pedicinidae, Pediculidae, and Pthirdae [29], and heteromyid rodents and *Fahrenholzia *lice [20,26,48]. Interestingly, this study finds that *Fahrenholzia *lice diverged approximately 31.6 Ma (95% HPD 49-16.6 Ma; Figure 3), a range that encompasses the divergence of their heteromyid hosts [48], further supporting cospeciation in this rodent-louse assemblage [26]. It is likely that additional instances of cospeciation will be revealed as more anopluran groups are examined. Given the lack of concordance between host and parasite trees (Figure 4), it is possible that anoplurans may have colonized their hosts and diversified after the initial radiation of eutherian mammals. Additional data and analyses will be necessary to test this possibility as all current data [this study and Smith VS, Ford T, Johnson KP, Johnson PCD, Yoshizawa K, Light JE: Multiple lineages of lice pass through the K-Pg boundary, Submitted] indicate a relatively old diversification of sucking lice.

## Conclusions

Anoplurans are one of only a handful of haematophagous arthropod groups, and as a result of their blood-feeding habits these ectoparastic insects can have severe effects on their hosts. Heavy infestations of sucking lice can cause host anemia, weight loss, damage to hides and fur (due to itching, scratching, and stains from louse feces), and general irritability, costing some livestock industries millions of dollars each year [7,49-51]. Sucking lice can also serve as vectors of pathogenic organisms, transmitting the causative agents for wildlife and livestock diseases such as swine pox, anaplasmosis, dermatomycosis, Lebombo virus, and seal heartworm [24]. Some anopluran species play important roles in their associations with humans. Head, body, and pubic lice (genera *Pediculus *and *Pthirus*, respectively) parasitize millions of people each year and *Pediculus *species are important in human epidemiology, serving as vectors of the causative agents of epidemic diseases such as trench fever (*Bartonella quintana*), relapsing fever (*Borrelia recurrentis*), and louse-borne typhus (*Rickettsia prowazekii*; [49]). Despite the potential economical and medical implications of sucking louse infestations, this study represents the first attempt to examine higher-level anopluran relationships using molecular data. Although this study produces novel findings regarding anopluran relationships, future studies with more extensive taxon sampling will be necessary to provide a better view of the evolutionary history of sucking lice.

Our analyses indicate that sucking lice diversified in the late Cretaceous, approximately 77 Ma, and soon after the Cretaceous-Paleogene boundary approximately 65 Ma, these lice proliferated rapidly to parasitize multiple mammalian orders and families (Figure 3). These dates are in agreement with mammalian evolutionary history: all modern mammal orders are hypothesized to have diverged by 75 Ma [20] thus providing suitable habitat for the colonization and radiation of sucking lice. Despite the concordant timing of diversification events early in the association between anoplurans and mammals, there is substantial conflict between the host and parasite phylogenies (Figure 4). This conflict may be because the free-living psocopteran ancestors of lice colonized and began feeding on the blood of their vertebrate hosts several times as host orders were diverging before the K-Pg boundary [20,52]. Additionally, host and parasite topological differences may be the result of a complex history of host switching and extinction events that occurred throughout the evolutionary association between sucking lice and their mammalian hosts. For example, multiple mammal lineages that radiated during the Early Tertiary (60-65 Ma) are now extinct as are their ectoparasitic lice (if present; although some of these lice may have successfully switched to an extant host). Furthermore, many of the modern mammal families did not diversify until recently, during the late Eocene to the Miocene (approximately 10-40 Ma), thus providing ample new habitats for lice to colonize.

It is unlikely that there are any ectoparasite groups (including lice) that tracked the early and rapid radiation of eutherian mammals (red and yellow boxes in Figure 4). This is in some ways unfortunate because ectoparasites could potentially be used as independent data points to infer host evolutionary history, something that could have been valuable to help elucidate remaining questions regarding the mammal phylogenetic tree [53,54]. Given the multiple host radiations and host extinction events [20], a combination of historical events such as extinction, host switching, and cospeciation likely dominated the evolutionary association between ectoparasites and their mammalian hosts. The sucking louse data presented herein support this scenario, and we expect that additional data from this group as well as other parasite lineages to further elucidate the complicated evolutionary history shared between parasites and their mammalian hosts.

## Methods

### Taxon Sampling and Data Collection

Data from 65 anopluran taxa (including 22 representatives from GenBank) representing 8 families and 48 species were included in the molecular analysis (Additional File 1). Seven outgroup taxa, representing the chewing louse suborders Rhynchophthirina and Ischnocera, also were included in the analysis. DNA was isolated from louse specimens using the DNAeasy Tissue Kit (QIAGEN Inc., Valencia, California) using louse specific protocols [11,55]. After DNA extraction, lice were mounted on slides and retained as vouchers. Voucher specimens are deposited in the Texas A&M University Insect Collection, voucher number 684.

Due to the large number of taxa obtained from previous studies, we focused our laboratory work on three genes that are well represented in GenBank. Portions of the nuclear 18S rRNA (18S; 460 or 508 base pairs [bp] depending on alignment methodology, see below) and elongation factor 1 alpha (EF-1α; 345 bp) genes and the mitochondrial cytochrome *c *oxidase subunit 1 (COI; 381 bp) gene were amplified and sequenced using primers NS1 and NS2a [12], EF1For3 and Cho10 [56], and L6625 and H7005 [57], respectively. Double-stranded PCR amplifications, PCR purification, and sequencing of these genes were undertaken following protocols detailed in Light and Reed [28]. Sequences were edited using Sequencher v. 4.2.2 (Gene Codes Corporation, Ann Arbor, Michigan) and primer sequences were removed and sequences trimmed in reference to the translated protein sequence using Se-AL v2.01a11 [58] and MacClade 4.0 [59]. The protein coding genes EF-1α and COI were aligned by eye using Se-Al v2.0a11 [58] and louse 18S rRNA sequences were aligned using CLUSTAL W [60] and MUSCLE [61]. Phylogenetic analyses of CLUSTAL W and MUSCLE alignments of 18S yielded similar topologies and branch lengths. Results presented herein rely solely on the MUSCLE 18S alignment (results based on CLUSTAL alignments are available upon request). Longer sequences obtained from GenBank were pruned for maximum overlap with sequences generated herein. All sequences are available in GenBank (Additional File 1) and alignments are available on TreeBase (http://purl.org/phylo/treebase/phylows/study/TB2:S10679; Submission ID 10669).

### Phylogenetic Analysis

Phylogenetic analyses of individual and combined genes were performed using maximum parsimony (MP), maximum likelihood (ML) and Bayesian approaches. Equally weighted MP searches were performed with 10 random addition replicates and tree bisection-reconnection branch swapping using PAUP*4.0b10 [62]. To assess nodal support, nonparametric bootstrap analyses were performed [200 pseudoreplicates and 10 random sequence additions; [63]]. To generate the best ML and Bayesian trees, Modeltest [64] and MrModelTest [65] were used to examine models of nucleotide substitution (56 and 24, respectively) and to choose a best-fit model of sequence evolution [66]. Models of evolution providing the best approximation of the data using the fewest parameters were chosen for subsequent analyses according to the Akaike Information Criterion [67,68]. The general time reversible (GTR) model, including among-site rate variation (G) and invariable sites [I; [69,70]] was chosen as the best model of evolution in both ModelTest and MrModelTest for the 18S rRNA, EF-1α (three codon positions combined and the third codon position), COI (three codon positions combined and the first and second codon positions), and the combined 3-gene data set. The GTR+G, SYM, and HKY+G models were chosen as the best model of evolution for the first EF-1α codon position, second EF-1α codon position, and third COI codon position, respectively. Full heuristic ML searches were conducted using the best-fit model in PAUP* 4.0b10 [62] and GARLI [71], and full heuristic ML bootstrap (100 pseudoreplicates) searches were conducted using the best-fit model in GARLI [71].

Bayesian phylogenetic analyses were performed in MrBayes 3.12 [72]. Model parameters were treated as unknown variables with uniform priors and were estimated as part of the analysis. Bayesian analyses were initiated from random starting trees, run for 10 million generations with 4 incrementally heated chains [72], and sampled at intervals of 1000 generations. Two independent Bayesian analyses were run to avoid entrapment on local optima, and log-likelihood scores were compared for convergence so that burn-in generations (the first 3000 trees) could be discarded. Tracer v1.4 [73] was used to evaluate stability of all parameter estimates following removal of burn-in generations.

The 3-gene data set also was examined with partitioned Bayesian phylogenetic analyses. Individual genes and codon positions were defined as partitions *a priori*, and each partition was assigned its own substitution model according to MrModelTest (see above). Partitioning schemes included non-partitioned, partitioned by gene, and partitioned by gene and by codon position of the protein coding genes COI and EF-1α. Partitioned Bayesian analyses were performed as described above. Bayesian partitioning schemes were compared using Bayes factors [74], which were computed using the harmonic means of the likelihoods calculated from the *sump *command within MrBayes. A difference of 2ln Bayes factor > 10 was used as the minimum value to discriminate between analysis schemes [30,75].

Bayesian Estimation of Species Trees [BEST; [76,77]] was used to simultaneously estimate gene and species trees while allowing for independent evolutionary processes for each locus. This type of approach is useful when multiple molecular markers are being used to infer a species tree and, if there are short branch lengths in the species tree, gene trees may not match the species tree resulting in incorrect inferences of species relationships [76,78]. Because missing data in individual gene trees can result in analytical complications [79], all taxa that were missing data from an entire gene region were removed prior to BEST analyses. To aid with computational time and to focus on the ingroup taxa, all outgroup taxa except for *Haematomyzus elephantis *were removed from the data set resulting in a total of 47 individuals analyzed. BEST analyses were initiated from random starting trees, run for 60 million generations and sampled at intervals of 1000 generations. The gene mutation prior and the prior distribution for the effective population size parameter were set at (0.5, 1.5) and 0.05, respectively. The posterior distribution of species trees (post burn-in) was summarized with a 50% majority-rule consensus tree to obtain posterior probability values for species relationships.

Alternative phylogenetic hypotheses were compared statistically using the Shimodaira-Hasegawa tests as implemented in PAUP*4.0b10 [80-82]. Additionally, suboptimal trees from the Bayesian non-partitioned and partitioned analyses were examined to assess alternative phylogenetic hypotheses. The frequency of the Markov chain Monte Carlo trees in agreement with an alternative hypothesis equals the probability of that alternative hypothesis being correct [83]. The probability of trees agreeing with alternative subfamily hypotheses was calculated by applying constraint-based filter trees implemented in PAUP*4.0b10 [83].

### Estimates of Divergence Times

Divergence times of the sucking louse 3-gene data set were estimated using the Bayesian approach implemented in BEAST v1.5.3 [31,36]. BEAST uses a Bayesian relaxed molecular clock while incorporating tree uncertainty in the MCMC process to infer divergence times. Before estimating divergence times, a likelihood ratio test was performed on the louse 3-gene data set using PAUP* 4.0b10 [62] to determine if the sequence data departed significantly from clocklike behavior. In BEAST, a Yule process speciation prior and an uncorrelated lognormal model of rate variation were implemented in each analysis [31]. Posterior probability distributions of node ages were obtained for the 3-gene alignment with analyses performed in a concatenated and partitioned framework (all model parameters were unlinked across partitions). Best-fit models of nucleotide substitution for each data set were the same as those identified above as part of the phylogenetic analyses using MrModelTest [65]. Two separate MCMC analyses were run for 30,000,000 generations (burnin 10%) with parameters sampled every 1000 steps. Independent runs were combined using LogCombiner v.1.5.3 [36]. Tracer v1.5 [73] was used to measure the effective sample size of each parameter (all resulting effective sample sizes exceeded 100) and calculate the mean and upper and lower bounds of the 95% highest posterior density interval (95% HPD) for divergence times. Tree topologies were assessed using TreeAnnotator v.1.5.3 [36] and FigTree v.1.3.1 [84]. Bayes factors of non-partitioned and partitioned 3-gene data sets were assessed using Tracer v1.5 [73].

Unfortunately, fossil calibrations for lice are lacking [10,17,18]. But, given their parasitic nature and the potential ancient origin of lice considering the recently described 100 Ma book louse fossil [Liposcelididae; [18]], it is reasonable to assume that sucking lice originated on their mammalian hosts by the end of the Cretaceous, 85-100 Ma [5,20]. Bininda-Emonds et al. [20] determined that time of basal diversification in placental mammals was 94-109 Ma; therefore, a calibration with a mean of 101 (and standard deviation of 3.5) was used to represent the basal split between the Rhychophthirina and Anoplura. Additionally, some studies have documented cospeciation (i.e., roughly contemporaneous speciation events) between sucking lice and their hosts, specifically primates and primate lice [29]. The sucking louse tree can therefore be calibrated by placing host fossil dates on the corresponding node of the louse tree. Corresponding to the split between Old World monkeys and apes [85], a calibration of 20-25 Ma was used to represent the split between Old World monkey lice (*Pedicinus spp*.) and hominoid-associated lice [29]. Furthermore, a calibration of 5-7 Ma (corresponding to the split between chimpanzees and humans) was used to represent the split between human *Pediculus *lice (*Pediculus humanus*) and the chimpanzee louse (*Pediculus schaeffi*; [86]). These three calibration points were used in combination, as well as individually, to cross-check the other calibration points, in the divergence dating analyses. Node constraints were assigned a normal prior distribution, with the standard deviations encompassing the minimum and maximum age of each calibration. Utilizing a normal distribution allows for uncertainty in the calibration estimates [87], which is important for our data because calibrations were taken from the host fossil record rather than from louse fossils.

## Authors' contributions

JEL conceived the study, carried out molecular studies and analyses, and wrote the manuscript. VSS and JMA collected molecular data and helped to draft the manuscript. LAD contributed samples to the study and helped to draft the manuscript. DLR participated in conceiving the study and drafting the manuscript. All authors read and approved the final manuscript.

## Supplementary Material

Additional file 1 **Louse taxa used in this study (arranged by family), host associations, and GenBank accession numbers**. Click here for file

Additional file 2 **Bayesian phylogram of the Anoplura based on the 18S rRNA gene**. Bayesian posterior probability greater than 0.95 are indicated above the nodes. Taxon names correspond to Additional File 1 and taxon colors correspond to louse family. Click here for file

Additional file 3 **Bayesian phylogram of the Anoplura based on the nuclear EF-1α gene**. Bayesian posterior probability greater than 0.95 are indicated above the nodes. Taxon names correspond to Additional File 1 and taxon colors correspond to louse family. This Bayesian phylogram is the result from a partitioned analysis with each codon position representing a distinct partition. Click here for file

Additional file 4 **Bayesian phylogram of the Anoplura based on the mitochondrial COI gene**. Bayesian posterior probability greater than 0.95 are indicated above the nodes. Taxon names correspond to Additional File 1 and taxon colors correspond to louse family. This Bayesian phylogram is the result from a partitioned analysis with each codon position representing a distinct partition. Click here for file

Additional file 5 **Chronogram for the Anoplura with the 95% highest posterior density interval indicated for each node**. Bayesian chronogram resulting from analysis of the 3-gene partitioned data set in BEAST. This figure is identical to Figure 3 with the exception of including the upper and lower bounds of the 95% highest posterior density interval (95% HPD) for each node. Click here for file
