# Supplementary material for: Evolutionary history of mammalian sucking lice (Phthiraptera: Anoplura)
Source: BMC Evol Biol. 2010 Sep 22;10:292. doi: 10.1186/1471-2148-10-292 (PMC2949877; doi:10.1186/1471-2148-10-292)
Supplement: Additional file 1 — Louse taxa used in this study (arranged by family), host associations, and GenBank accession numbers. [file 1471-2148-10-292-S1.DOC]

Additional File 1. Louse taxa used in this study (arranged by family), host associations, and GenBank accession numbers.

Louse Family and Species (Locality) Taxon Label Host (Order: Family; Museum Voucher) 18S EF1 COI

|  |  |  |  |  |  |
| --- | --- | --- | --- | --- | --- |
| **Echinophthiriidae** |  |  |  |  |  |
| *Echinophthirius horridus* (Unknown) | 069_*Echin_horridus_b* | *Phoca vitulina* (Carnivora: Phocidae) | HM171377 | AF320396 | HM171455 |
| *Proechinophthirus fluctus* (USA: AK) | Echin3.17.09.2 | *Callorhinus ursinus* (Carnivora: Otariidae) | HM171378 | HM171456 | HM171421 |
|  |  |  |  |  |  |
| **Haematopinidae** |  |  |  |  |  |
| *Haematopinus eurysternus* (Egypt) | Hpeur2.16.2008.5 | *Bos* sp. (Artiodactyla: Bovidae) | HM171381 | HM171457 | HM171422 |
| *Haematopinus phacochoeri* (Unknown) | 076_*Haem_phacochoeri* | *Phacochoerus aethiopicus* (Artiodactyla: Suidae) | AF385058 | AF385026 | AF385007 |
| *Haematopinus suis* (Unknown) | 074_*Haem_suis* | *Sus scrofa* (Artiodactyla: Suidae) | HM171379 | EU375777 | EU375756 |
| *Haematopinus tuberculatus* (Unknown) | 075_*Haem_tuberculatus* | *Bubalus bubalis* (Artiodactyla: Bovidae) | HM171380 | EU375778 | EU375757 |
|  |  |  |  |  |  |
| **Hoplopleuridae** |  |  |  |  |  |
| *Ancistroplax crocidurae* 1 (Vietnam) | Axcro4.26.09.1 | *Crocidura* sp. (Soricomorpha: Soricidae) | HM171382 | X | HM171423 |
| *Ancistroplax crocidurae* 2 (China) | Axsp9.23.2008.4 | *Crocidura attenuata* (Soricomorpha: Soricidae) | HM171383 | HM171458 | HM171424 |
| *Hoplopleura aitkeni* (Peru: Cusco) | *Hop_aitkeni*_174952 | *Akodon aerosus* (Rodentia: Cricetidae; FMNH 174952) | X | EU375787 | EU375769 |
| *Hoplopleura arizonensis* (USA: AZ) | Hoari1.31.2008.4 | *Sigmodon fulviventer* (Rodentia: Cricetidae; NMMNH 4409) | HM171384 | HM171459 | HM171425 |
| *Hoplopleura* *erratica* (USA: MI) | Hosp.8.12.2008.2 | *Tamias* *striatus* (Rodentia: Sciuridae; LSUMZ 36349) | HM171385 | HM171460 | HM171426 |
| *Hoplopleura ferrisi* 1 (MX: Puebla) | Hofer4.16.2008.4 | *Peromyscus difficilis* (Rodentia: Cricetidae; LSUMZ 36246) | HM171386 | HM171461 | X |
| *Hoplopleura ferrisi* 2 (MX: Puebla) | Hofer11.17.2008.3 | *Peromyscus difficilis* (Rodentia: Cricetidae; LSUMZ 36247) | HM171387 | HM171462 | HM171427 |
| *Hoplopleura ferrisi* 3 (MX: Mexico) | Hofer7.30.2008.1 | *Peromyscus melanotis* (Rodentia: Cricetidae; CNMA 41034) | HM171388 | HM171463 | HM171428 |
| *Hoplopleura fonsecai* (Peru: Madre de Dios) | *Hop_fonsecai*_175207 | *Oxymycterus inca* (Rodentia: Cricetidae; FMNH 175207) | X | EU375788 | EU375770 |
| *Hoplopleura hesperomydis* (USA: CA) | Hohes3.29.2008.3 | *Peromyscus maniculatus* (Rodentia: Cricetidae; MLZ 1861) | HM171389 | HM171464 | HM171429 |
| *Hoplopleura hirsuta* (USA: TX) | Hosp.4.16.2008.5 | *Sigmodon hispidus* (Rodentia: Cricetidae; LSUMZ 36377) | HM171390 | HM171465 | HM171430 |
| *Hoplopleura* new sp. (Peru: Cusco) | *Hop_*newsps2_170689 | *Thomasomys aureus* (Rodentia: Cricetidae; FMNH 170689) | X | EU375797 | EU375776 |
| *Hoplopleura onychomydis* (USA: AZ) | Hoony1.31.2008.3 | *Onychomys torridus* (Rodentia: Cricetidae; NMMNH 4394) | HM171391 | HM171466 | HM171431 |
| *Hoplopleura quadridentata* (Peru: Cusco) | *Hop_quadridentata_*172246 | *Neacomys* sp. (Rodentia: Cricetidae; FMNH 17224) | X | EU375792 | EU375771 |
| *Hoplopleura reithrodontomydis* 1 (USA: AZ) | Hosp8.12.2008.4 | *Reithrodontomys* sp. (Rodentia: Cricetidae; NMMNH 4410) | HM171392 | HM171467 | HM171432 |
| *Hoplopleura reithrodontomydis* 2 (USA: AZ) | Hosp11.17.2008.1 | *Reithrodontomys* sp. (Rodentia: Cricetidae; NMMNH 4411) | HM171393 | HM171468 | HM171433 |
| *Hoplopleura rimae* (Peru: Cusco) | *Hop_rimae*_170569 | *Microryzomys minutus* (Rodentia: Cricetidae; FMNH 170569) | X | EU375793 | EU375772 |
| *Hoplopleura sicata* (China) | Hosic7.30.2008.3 | *Niviventer fulvescens* (Rodentia: Muridae) | HM171394 | HM171469 | HM171434 |
| *Hoplopleura tiptoni* (Peru: Cusco) | *Hop_tiptoni*_175243 | *Thomasomys oreas* (Rodentia: Cricetidae; FMNH 175243) | X | EU375796 | EU375775 |
| *Hoplopleura travassosi* (Peru: Cusco) | *Hop_travassosi*_175102 | *Oligoryzomys destructor* (Rodentia: Cricetidae; FMNH 175102) | X | EU375794 | EU375773 |
| *Hoplopleura trispinosa* (Unknown) | 082_*Hoplopleura_trispinosa* | *Glaucomys volans* (Rodentia: Sciuridae) | HM171395 | HM171470 | HM171435 |
| *Pterophthirus imitans* (Peru: Cusco) | 083_*Pterophthirus_imitans* | *Cavia aperea* (Rodentia: Caviidae) | HM171396 | EU375781 | EU375762 |
| *Pterophthirus splendida* (Peru: Madre de Dios) | *Pter_splendida_*170726 | *Proechimys simonsi* (Rodentia: Echimyidae) | X | EU375782 | EU375763 |
|  |  |  |  |  |  |
| **Linognathidae** |  |  |  |  |  |
| *Linognathus africanus* (Unknown) | 072_*Linognathus_africanus* | *Capra hircus* (Artiodactyla: Bovidae) | HM171397 | X | EU375760 |
| *Linognathus ovillus* (Unknown) | 070_*Linognathus_ovillus* | *Ovis aries* (Artiodactyla: Bovidae) | HM171398 | X | EU375761 |
| *Linognathus spicatus* (Zimbabwe) | Linog6.22.09.1 | *Connochaetes taurinus* (Artiodactyla: Bovidae) | HM171399 | HM171471 | HM171436 |
|  |  |  |  |  |  |
| Additional File 1. Continued |  |  |  |  |  |
|  |  |  |  |  |  |
| **Pedicinidae** |  |  |  |  |  |
| *Pedicinus badii* 1 (Uganda) | Qnbad7.24.06.9 | *Piliocolobus tephrosceles* (Primates: Cercopithecidae) | FJ267403 | EF152563 | EF152556 |
| *Pedicinus badii* 2 (Uganda) | Qnbad7.24.06.8 | *Piliocolobus rufomitratus* (Primates: Cercopithecidae) | X | HM171472 | HM171437 |
| *Pedicinus badii* 3 (Uganda) | Qnsp10.09.09.2 | *Piliocolobus rufomitratus* (Primates: Cercopithecidae) | HM171400 | HM171473 | HM171438 |
| *Pedicinus hamadryas* (USA: captive) | Qnham2.4.01.2 | *Papio hamadryas* (Primates: Cercopithecidae) | HM171401 | EU152562 | AY696007 |
| *Pedicinus pictus* 1 (Ivory Coast) | Qnpic3.31.08.1 | *Piliocolobus badius* (Primates: Cercopithecidae) | HM171402 | HM171474 | HM171439 |
| *Pedicinus pictus* 2 (Ivory Coast) | Qnpic6.30.09.2 | *Colobus polykomos* (Primates: Cercopithecidae) | X | X | HM171440 |
| *Pedicinus pictus* 3 (Ivory Coast) | Qnsp3.31.08.3 | *Colobus polykomos* (Primates: Cercopithecidae) | HM171403 | X | HM171441 |
|  |  |  |  |  |  |
| **Pediculidae** |  |  |  |  |  |
| *Pediculus humanus capitis* 3 (USA: UT) | Pdcap3.27.07.3WW | *Homo sapiens* (Primates: Hominidae) | FJ267396 | FJ267439 | EU493445 |
| *Pediculus humanus capitis* 20 (USA: FL) | Pdcap9.20.05.20WW | *Homo sapiens* (Primates: Hominidae) | FJ267397 | FJ267440 | EU493446 |
| *Pediculus humanus capitis* 23 (USA: FL) | Pdcap9.20.05.23NW | *Homo sapiens* (Primates: Hominidae) | FJ267394 | FJ267438 | EU493447 |
| *Pediculus humanus capitis* 25 (USA: FL) | Pdcap9.20.05.25NW | *Homo sapiens* (Primates: Hominidae) | FJ267395 | EF152558 | EF152552 |
| *Pediculus humanus humanus* (Burundi) | Pdhum9.6.06.3BurB | *Homo sapiens* (Primates: Hominidae) | FJ267399 | FJ267441 | FJ267426 |
| *Pediculus schaeffi* (Uganda) | Pdsch5.23.05.4 | *Pan troglodytes* (Primates: Hominidae) | FJ267400 | EF152559 | EF152553 |
|  |  |  |  |  |  |
| **Polyplacidae** |  |  |  |  |  |
| *Fahrenholzia ehrlichi* 1 (USA: TX) | Fzehr3.9.2008.3 | *Liomys irroratus* (Rodentia: Heteromyidae; LSUMZ 36395) | HM171404 | X | HM171442 |
| *Fahrenholzia ehrlichi* 2 (MX: Puebla) | Fzehr4.16.2008.2 | *Liomys irroratus* (Rodentia: Heteromyidae; LSUMZ 36299) | HM171405 | HM171475 | HM171443 |
| *Fahrenholzia pinnata* (USA: NV) | Fzpin163.1 | *Perognathus longimembris* (Rodentia: Heteromyidae; MLZ 2039) | FJ267392 | EF152563 | EF152557 |
| *Fahrenholzia reducta* 1 (USA: CA) | Fzred8.12.2008.3 | *Chaetodipus formosus* (Rodentia: Heteromyidae; MLZ 1865) | HM171406 | HM171476 | HM171444 |
| *Fahrenholzia reducta* 2 (USA: CA) | Fzred7.24.06.10 | *Chaetodipus formosus* (Rodentia: Heteromyidae; MLZ 1863) | FJ267393 | FJ267437 | FJ267424 |
| *Fahrenholzia zacatecae* (USA: NM) | Fzzac11.17.2009.2 | *Chaetodipus eremicus* (Rodentia: Heteromyidae; NMMNH 4435) | HM171407 | HM171477 | HM171445 |
| *Haemodipsus brachylagi* (USA: NV) | Habra9.4.08.6 | *Brachylagus idahoensis* (Lagomorpha: Leporidae) | HM171408 | HM171478 | HM171446 |
| *Lemurpediculus verruculosus* 1 (Madagascar) | Lesp2.3.2008.4 | *Microcebus rufus* (Primates: Cheirogaleidae) | HM171409 | HM171479 | HM171447 |
| *Lemurpediculus verruculosus* 2 (Madagascar) | Lever4.26.09.2 | *Microcebus rufus* (Primates: Cheirogaleidae) | HM171410 | HM171480 | HM171448 |
| *Linognathoides laeviusculus* (USA: AK) | Lnlae1.31.2008.6 | *Spermophilus parryii* (Rodentia: Sciuridae; UAM 72218) | HM171411 | X | HM171449 |
| *Linognathoides marmotae* 1 (USA: CO) | Lnlae6.30.09.3 | *Marmota flaviventris* (Rodentia: Sciuridae) | HM171412 | HM171481 | HM171450 |
| *Linognathoideas marmotae* 2 (Unknown) | 088_*Lin_marmotae* | *Marmota* sp. (Rodentia: Sciuridae) | HM171413 | EU375780 | EU375789 |
| *Neohaematopinus neotomae* 1 (USA: NM) | Neneo4.16.2008.6 | *Neotoma albigula* (Rodentia: Cricetidae; NMMNH 3930) | HM171414 | HM171482 | HM171451 |
| *Neohaematopinus neotomae* 2 (USA: CA) | Neneo1.31.2008.2 | *Neotoma lepida* (Rodentia: Cricetidae; MLZ 1921) | HM171415 | HM171483 | X |
| *Neohaematopinus sciuropteri* (USA: OR) | Nescp4.16.2008.1 | *Glaucomys sabrinus* (Rodentia: Sciuridae) | HM171416 | HM171484 | HM171452 |
| *Neohaematopinus sciuri* (Unknown) | 085_*Neo_sciuri* | *Sciurus carolinensis* (Rodentia: Sciuridae) | AF385060 | AF320433 | AF385008 |
| *Polyplax serrata* (Czech Republic) | *Polyplax_serrata*_CB1As | *Apodemus sylvaticus* (Rodentia: Muridae) | X | EU162272 | EU162163 |
| *Sathrax durus* (Vietnam) | Sathrax4.26.09.3 | *Tupaia belangeri* (Scandetia: Tupaiidae) | HM171417 | HM171485 | HM171453 |
|  |  |  |  |  |  |
| **Pthiridae** |  |  |  |  |  |
| *Pthirus gorillae* (Uganda) | Ptgor8.1.06.1 | *Gorilla gorilla* (Primates: Hominidae) | FJ267402 | EF152561 | EF152555 |
| *Pthirus* *pubis* (Scotland) | Ptpub1.19.06.3 | *Homo sapiens* (Primates: Hominidae) | FJ267401 | EF152560 | EF152554 |
|  |  |  |  |  |  |
|  |  |  |  |  |  |
|  |  |  |  |  |  |
|  |  |  |  |  |  |
|  |  |  |  |  |  |
|  |  |  |  |  |  |
| Additional File 1. Continued |  |  |  |  |  |
|  |  |  |  |  |  |
| **Rhynchophthirina (Outgroup)** |  |  |  |  |  |
| *Haematomyzus elephantis* (India) | 059_*Haem_elephantis* | *Elephas maximus* (Proboscidea: Elephantidae) | AY077778 | AF320405 | AY314816 |
|  |  |  |  |  |  |
| **Ischnocera (Outgroup)** |  |  |  |  |  |
| *Bovicola* sp. | 026_*Bovicola_sp.* | *Ovis aries* (Artiodactlya: Bovidae) | AY077769 | AF320370 | AF545680 |
| *Docophoroides brevis* | 054_*Doco_brevis* | *Diomedea exulans* (Procellariiformes: Diomedeidae) | HM171418 | AF320394 | AF396547 |
| *Felicola subrostratus* | 028_*Feli_subrostratus* | *Felis catus* (Carnivora: Felidae) | HM171419 | AF320398 | HM171454 |
| *Columbicola columbae* | 037_*Col_columbae* | *Columba livia* (Columbiformes: Columbidae) | AF385044 | AF320385 | AF278620 |
| *Neotrichodectes* sp. | 030_*Neotrichodectes_sp.* | Uncertain | HM171420 | A545794 | AF545736 |
| *Pectinopygus sulae* | 036a_*Pectinopygus_sulae* | *Sula sula* (Pelecaniformes: Suidae) | AY077768 | AF320444 | AY314804 |

Museum acronyms for host taxa installed in Natural History Museums are as follows: Colección Nacional de Mamíferos, Universidad Nacional Autónoma de México (CNMA), Field Museum of Natural History (FMNH) Louisiana State University Museum of Natural Science (LSUMZ), Moore Laboratory of Zoology, Occidental College (MLZ), New Mexico Museum of Natural History (NMMNH), and University of Alaska Museum of the North (UAM). GenBank accessions listed as ‘X’ denotes missing data.
