# Supplementary figures and images for: Evolutionary history of mammalian sucking lice (Phthiraptera: Anoplura)
Source: BMC Evol Biol. 2010 Sep 22;10:292. doi: 10.1186/1471-2148-10-292 (PMC2949877; doi:10.1186/1471-2148-10-292)

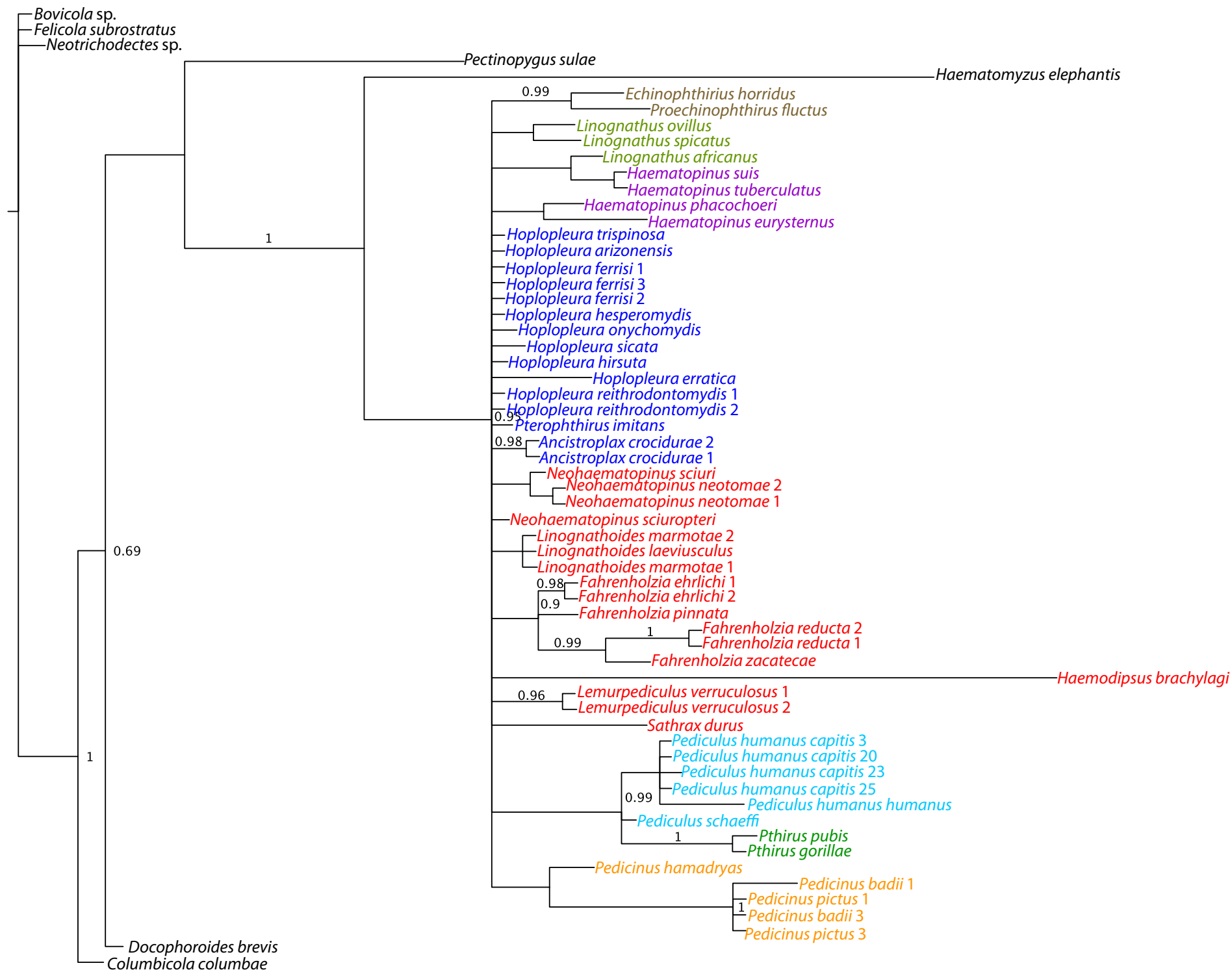

Supplement: Additional file 2 — Bayesian phylogram of the Anoplura based on the 18S rRNA gene. Bayesian posterior probability greater than 0.95 are indicated above the nodes. Taxon names correspond to Additional File 1 and taxon colors correspond to louse family. [file 1471-2148-10-292-S2.PDF]

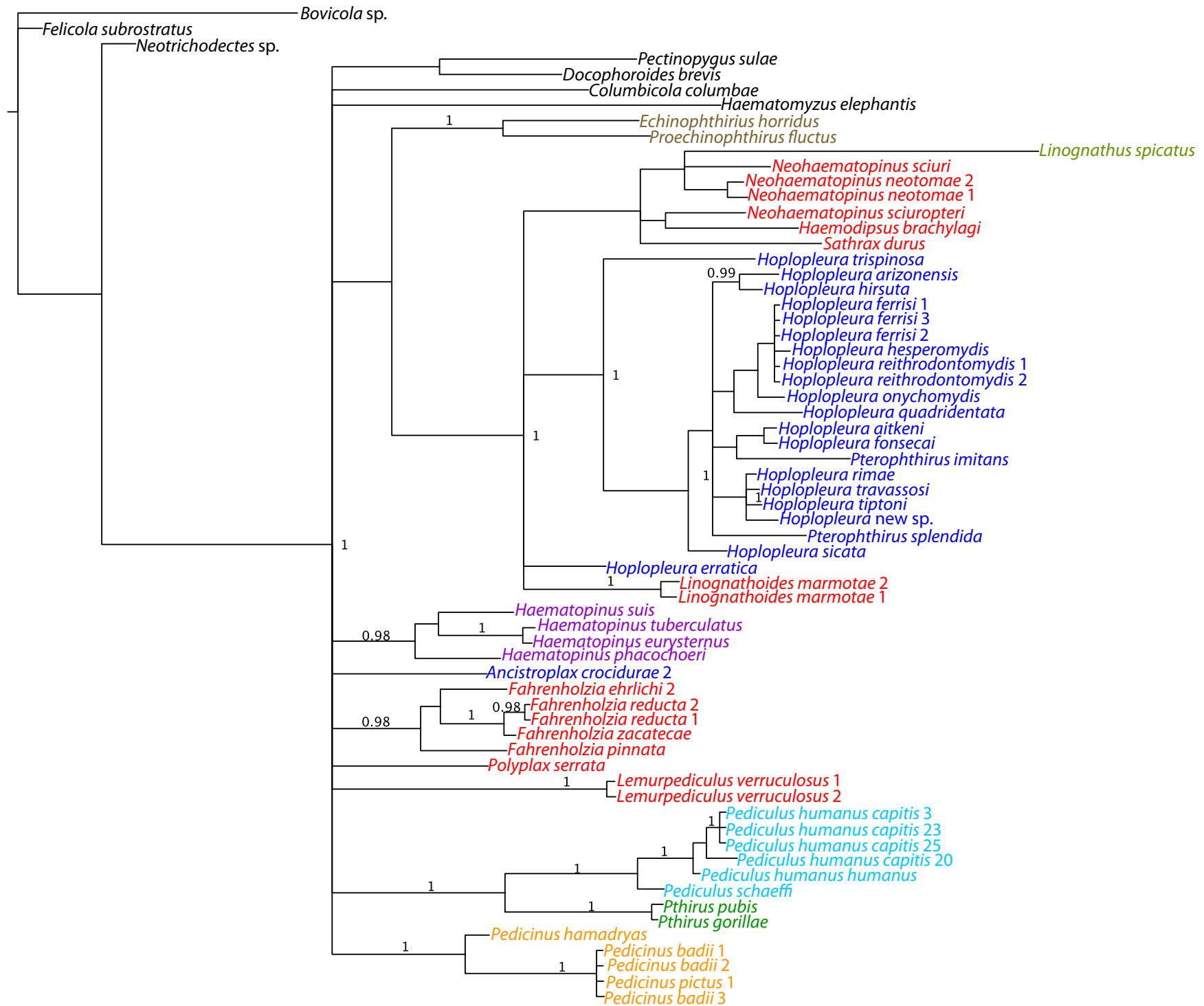

Supplement: Additional file 3 — Bayesian phylogram of the Anoplura based on the nuclear EF-1α gene. Bayesian posterior probability greater than 0.95 are indicated above the nodes. Taxon names correspond to Additional File 1 and taxon colors correspond to louse family. This Bayesian phylogram is the result from a partitioned analysis with each codon position representing a distinct partition. [file 1471-2148-10-292-S3.PDF]

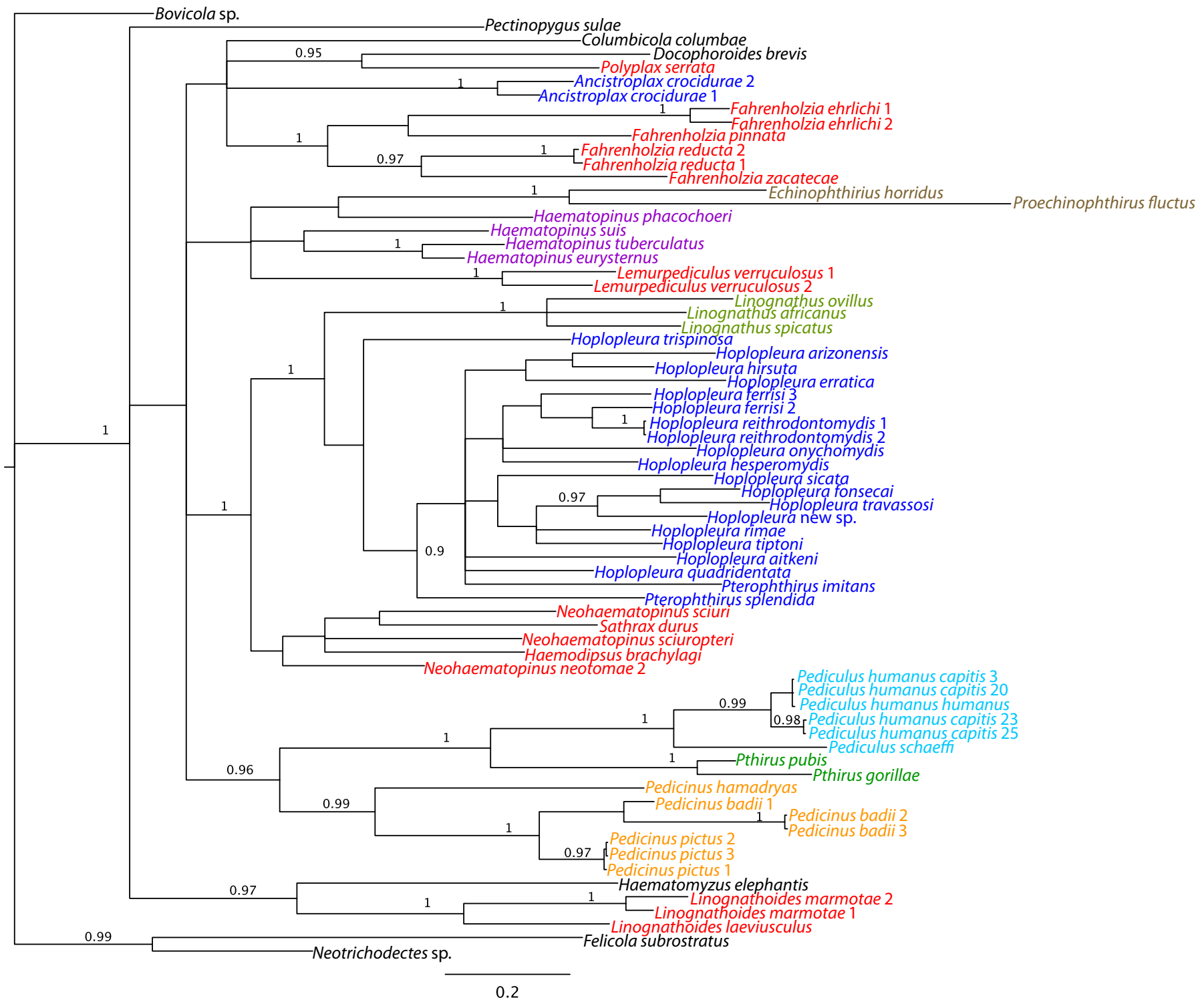

Supplement: Additional file 4 — Bayesian phylogram of the Anoplura based on the mitochondrial COI gene. Bayesian posterior probability greater than 0.95 are indicated above the nodes. Taxon names correspond to Additional File 1 and taxon colors correspond to louse family. This Bayesian phylogram is the result from a partitioned analysis with each codon position representing a distinct partition. [file 1471-2148-10-292-S4.PDF]

K-Pg  
(65 Ma)

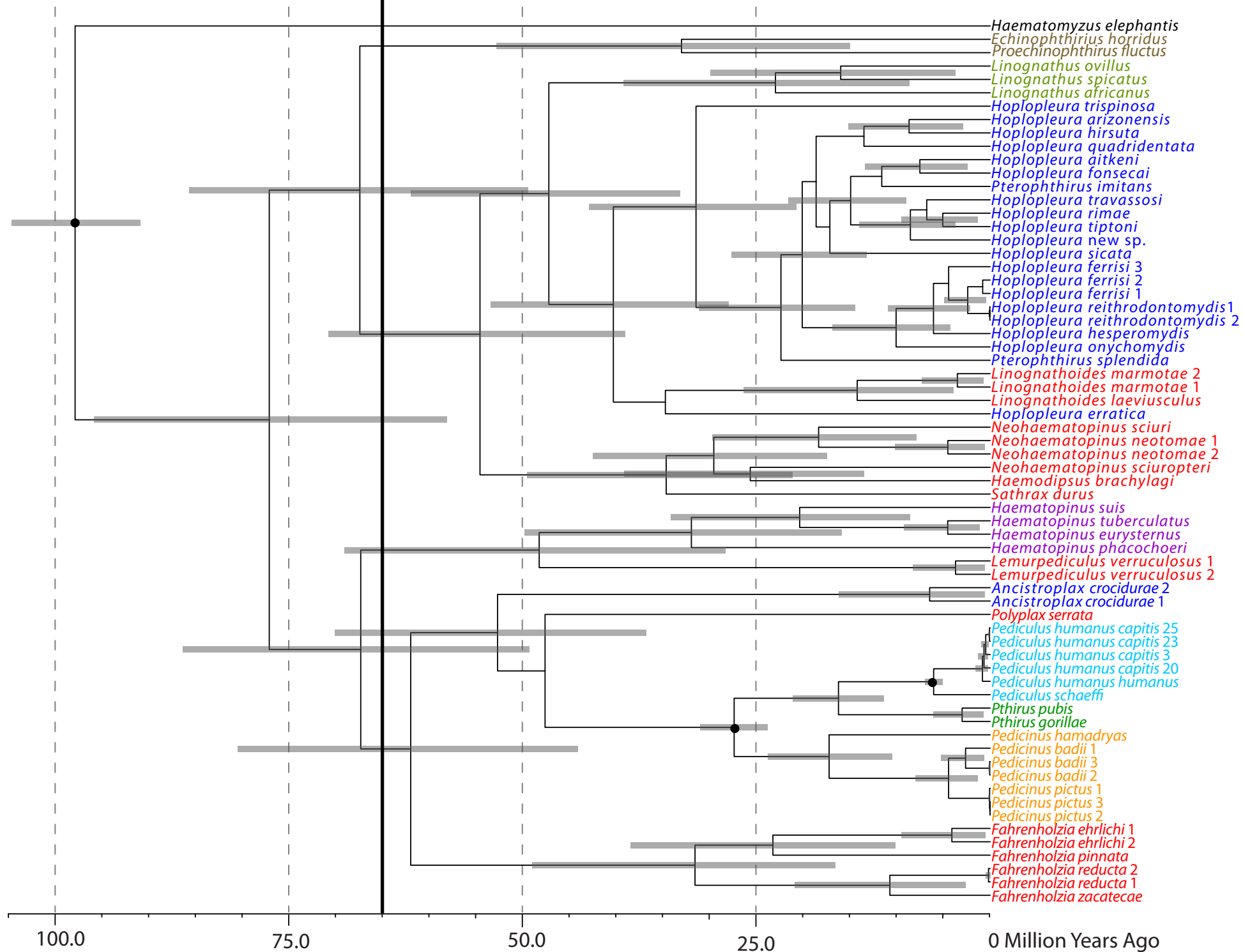

Supplement: Additional file 5 — Chronogram for the Anoplura with the 95% highest posterior density interval indicated for each node. Bayesian chronogram resulting from analysis of the 3-gene partitioned data set in BEAST. This figure is identical to Figure 3 with the exception of including the upper and lower bounds of the 95% highest posterior density interval (95% HPD) for each node. [file 1471-2148-10-292-S5.PDF]
